# Alkaline Stability of Anion-Exchange Membranes

**DOI:** 10.1021/acsaem.2c03689

**Published:** 2023-01-09

**Authors:** Sapir Willdorf-Cohen, Avital Zhegur-Khais, Julia Ponce-González, Saja Bsoul-Haj, John R. Varcoe, Charles E. Diesendruck, Dario R. Dekel

**Affiliations:** † The Wolfson Department of Chemical Engineering, Technion—Israel Institute of Technology, Haifa3200003, Israel; ‡ School of Chemistry and Chemical Engineering, University of Surrey, GuildfordGU2 7XH, U.K.; § Schulich Faculty of Chemistry, Technion—Israel Institute of Technology, Haifa3200003, Israel; ∥ The Nancy & Stephen Grand Technion Energy Program (GTEP), Technion—Israel Institute of Technology, Haifa3200003, Israel

**Keywords:** anion-exchange membranes, fuel cells, water electrolyzers, functional groups, alkaline stability, chemical degradation

## Abstract

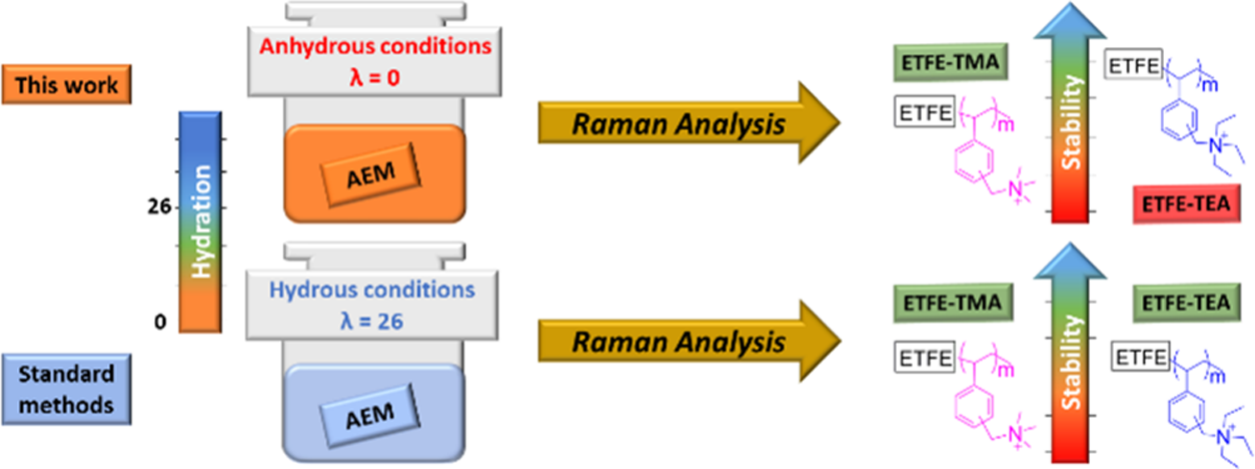

Recently, the development of durable anion-exchange membrane fuel cells (AEMFCs) has increased in intensity due to their potential to use low-cost, sustainable components. However, the decomposition of the quaternary ammonium (QA) cationic groups in the anion-exchange membranes (AEMs) during cell operation is still a major challenge. Many different QA types and functionalized polymers have been proposed that achieve high AEM stabilities in strongly alkaline aqueous solutions. We previously developed an *ex situ* technique to measure AEM alkaline stabilities in an environment that simulates the low-hydration conditions in an operating AEMFC. However, this method required the AEMs to be soluble in DMSO solvent, so decomposition could be monitored using ^1^H nuclear magnetic resonance (NMR). We now report the extension of this *ex situ* protocol to spectroscopically measure the alkaline stability of insoluble AEMs. The stability ofradiation-grafted (RG) poly(ethylene-*co*-tetrafluoroethylene)-(ETFE)-based poly(vinylbenzyltrimethylammonium) (ETFE-TMA) and poly(vinylbenzyltriethylammonium) (ETFE-TEA) AEMs were studied using Raman spectroscopy alongside changes in their true OH^–^ conductivities and ion-exchange capacities (IEC). A crosslinked polymer made from poly(styrene-*co*-vinylbenzyl chloride) random copolymer and *N*,*N*,*N*′,*N*′-tetraethyl-1,3-propanediamine (TEPDA) was also studied. The results are consistent with our previous studies based on QA-type model small molecules and soluble poly(2,6-dimethylphenylene oxide) (PPO) polymers. Our work presents a reliable *ex situ* technique to measure the true alkaline stability of AEMs for fuel cells and water electrolyzers.

## Introduction

In recent years, anion-exchange membrane fuel cells (AEMFCs) and electrolyzers (AEMWEs) have been attracting growing attention due to their potential to reduce the requirement for high-cost, unsustainable components (catalysts, bipolar plates), leading to easier future commercialization of fuel cell and water electrolyzer applications.^1−9^ Anion-exchange membranes (AEMs) utilize immobilized cationic groups, typically quaternary ammonium (QAs) groups,^10−16^ to enable conduction of the OH^–^ ions through the AEM of the AEMFC or AEMWE. Unfortunately, QA groups can decompose under the relatively low-hydration alkaline environment of the electrochemical devices, causing a decrease in cell performance due to a decrease in AEM conductivity and poorer water transport, limiting the device’s lifetime.^17−19^ Although newly developed AEMs often show modest improvements in *ex situ* stability tests, they still commonly undergo rapid *operando* decomposition.^6,20−24^

Aiming to overcome these stability issues, several polymeric backbones have been synthesized and reported in the literature, such as polyethersulfone, polyfluoro-olefins, and poly(norbornene).^25−27^ In addition, most research into AEM stability improvements focused on developing of new QA chemistries.^16,28−34^ AEMs containing benzyltrimethylammonium (BTMA), the most commonly studied QA, can, however, exhibit decent alkaline stability in AEM form, especially when fully hydrated.^35−38^ Currently, the main issue is the lack of test condition standardization: most alkaline stability testing is carried out at extremely high hydration, *e.g*., λ > 50 (λ = water molecules/OH^–^ molecules).^39−42^ Even if an *ex situ* stability test is conducted with concentrated aqueous solutions (up to 10 M),^43−45^ the high number of water molecules around the OH^–^ anions (λ ≥ 6) may cause the AEM to appear stable, while actually be unstable in *operando* tests. Thus, the presence of water molecules in the first solvation sphere of the OH^–^ anions is a major parameter affecting AEMs stability.^17,35,46−48^ In an AEMFC operated under practical current densities (≥400 mA cm^–2^) or AEMWE (in dry cathode operation mode),^49,50^ the water consumption at the cathode side is highly increased, and microsolvation of OH^–^ anions is reduced to very low levels (*ca*. λ = 2).^51^ Due to this low hydration level, the undersolvated OH^–^ ions are highly aggressive nucleophiles.^35,46^ This can explain why QA groups (especially in cathode ionomers and in cathode facing sides of AEMs) can rapidly degrade in *operando* AEMFC tests, even if the same QA groups demonstrate high alkaline resistance in *ex situ* stability tests involving aqueous solutions.

In our previous studies, we demonstrated an effective, novel *ex situ* protocol for monitoring the degradation of QA groups in 0.6 M OH^–^ concentration environments containing strictly controlled water contents, which demonstrated that the decomposition of such QA salts is extremely sensitive to water content, particularly at λ < 5 levels, with rate constants spanning several orders of magnitude.^46,52^ We also showed that unstable QA model compounds could be stabilized using sufficient λ values (8–10).^48^ So far, our protocol (using ^1^H NMR measurements) has allowed a fundamental understanding of the degradation kinetics of soluble BTMA and benzyltriethylammonium (BTEA) salts, and also when such chemistries were covalently attached to DMSO-soluble linear poly(2,6-dimethylphenylene oxide) (PPO). It was observed that the degradation rate constants for the soluble polymers were *ca*. an order of magnitude higher compared to the small molecule analogues, indicating that the covalent attachment of QA groups onto a polymer chain had a negative effect on stability.^35,46^ Our *ex situ* alkali stability protocol for soluble AEMs and model compounds provided results that better mimic the environment of the electrochemical device in operation.

Recently reported radiation-grafted AEMs (RG-AEMs) and thin, crosslinked aliphatic AEMs have shown significant improvements in AEMFC performance.^12,37,53−57^ These types of AEMs are useful for alkaline stability insights as they can be fabricated with a wealth of alterable experimental parameters (*e.g*., same chemistry and thickness but different ion-exchange capacities (IEC), or same IEC and thickness but different chemistries).^53^ However, they are totally insoluble, leading to an essential requirement for a consistent, meaningful *ex situ* stability test protocol that can be applied to insoluble AEMs.

Here, we expand our protocol to investigate the alkali stability of both RG-AEMs (either containing BTMA or BTEA groups) and crosslinked AEMs (neither being soluble in solvents under non-extreme conditions) and follow the alkaline decomposition kinetics using spectroscopic methods. The results are verified by measuring the changes in IECs and true OH^–^ conductivities.^58^ This study provides a better understanding of the water microsolvation effect on the degradation of solid-state AEMs.

## Experimental Section

### General

*N*,*N*,*N*′,*N*′-Tetraethyl-1,3-propanediamine (TEPDA, 97%), vinylbenzyl chloride (VBC), and anhydrous DMSO (≥99.9%) were purchased from Sigma-Aldrich. Styrene (St) and 2,2,6,6-tetramethyl-1-piperidinyloxy (TEMPO) were purchased from Alfa Aesar. KOH and benzoyl peroxide (BPO) were acquired from Bio-Lab and Merck, respectively. All chemicals were used without further purification unless noted. Stability tests at low hydration level (λ = 0) were performed in an MBraun MS-Unilab Pro SP glovebox, with a room-temperature nitrogen atmosphere containing less than 0.1 ppm water and oxygen. Gel permeation chromatography (GPC) analysis was done in THF at 30 °C, according to the polymer’s solubility, using a Thermo LC system. The THF system consisted of one Tosoh TSKgel HHR-L guard column and four TSKgel G4000HHR columns in sequence, working at a 1 mL min^–1^ flow rate.

### Preparation of the VBC-grafted ETFE

The procedure for preparing RG-AEMs is described in detail elsewhere.^59^ Briefly, the ETFE precursor film (25 μm thick) is first pre-irradiated in air (peroxidated using an electron beam with a total absorbed dose of 30 kGy) and then grafted with VBC monomer (3- and 4-isomer mix), yielding poly(VBC)-grafted ETFE intermediate film. There is no consensus whether the grafted poly(VBC) chains are attached to the ETFE substrate chains *via* an oxygen atom or directly *via* a C–C bond.^60^

### ETFE-Based RG-AEM Containing BTMA Group (ETFE-TMA)

For the amination step, the poly(VBC)-grafted ETFE intermediate film was immersed in aqueous trimethylamine (TMA) solution (45% w/w) and stirred at room temperature for 24 h.^59^ The resultant ETFE-TMA AEM had a measured hydrated thickness of 50 μm and an IEC = 1.65 mmol g^–1^ (Cl^–^ form).

### ETFE-Based RG-AEM Containing BTEA Group (ETFE-TEA)

**Figure 1:**
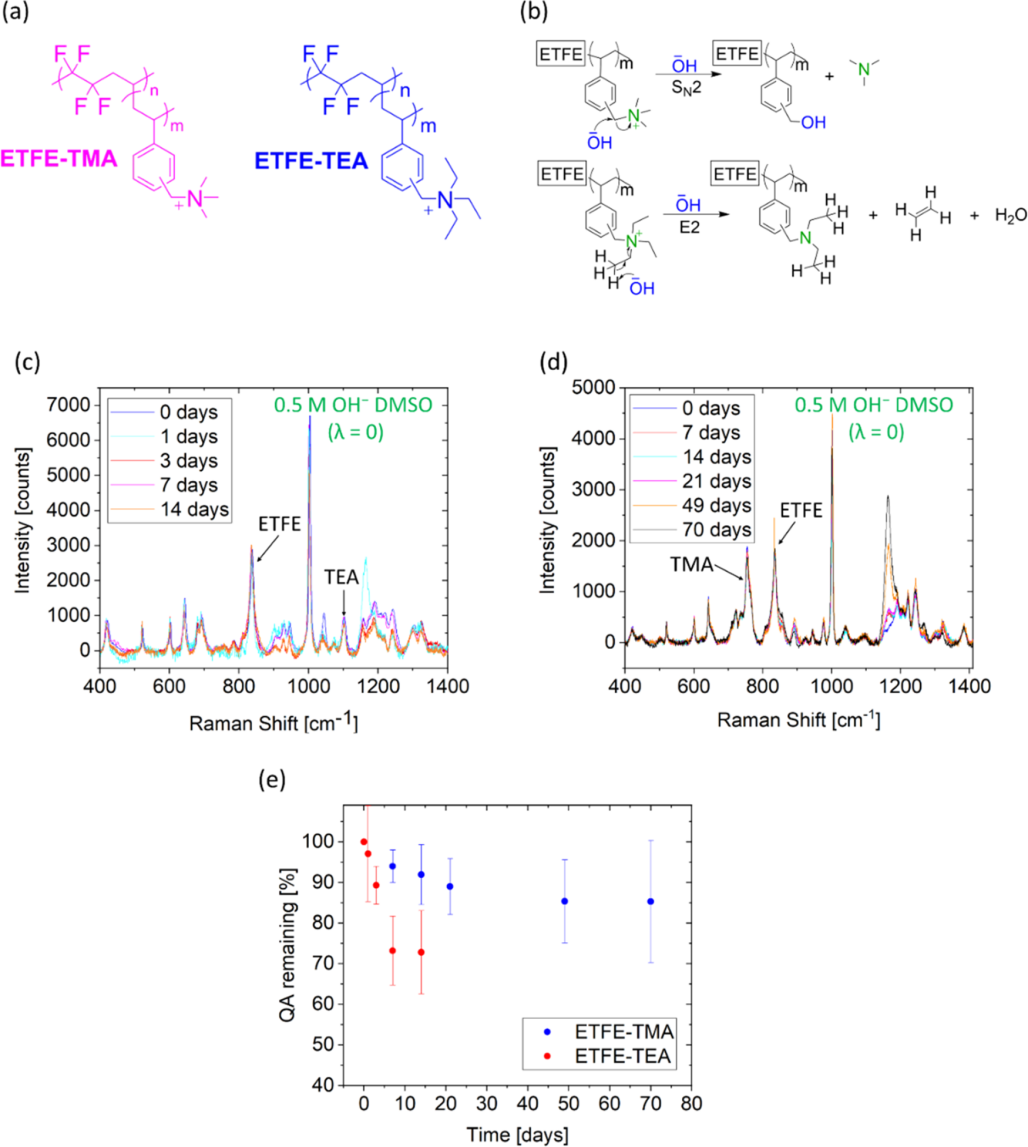
(a) Chemical structures of ETFE-TMA and ETFE-TEA. (b) Degradation mechanisms of ETFE-TMA by nucleophilic attack (S_N_2) and ETFE-TEA by Hofmann elimination (E2). Raman spectra of (c) ETFE-TEA and (d) ETFE-TMA immersed in 0.5 M OH^–^ DMSO (λ = 0) solution. (e) QA decay in ETFE-TMA and ETFE-TEA as a function of time measured by Raman, when tested in 0.5 M OH^–^ DMSO solutions (λ = 0). Error bars represent sample standard deviations from three measurements.

An ETFE-TEA AEM, Figure 1, was prepared in a similar manner as the ETFE-TMA, but the poly(VBC)-grafted ETFE intermediate film was immersed in undiluted triethylamine and heated to 80 °C for 5 days. ETFE-TEA had a measured hydrated thickness of *ca*. 50 μm and an IEC of 1.70 mmol g^–1^ (Cl^–^ form).

### Preparation of P(St-*co*-VBC)

The procedure for synthesis was adapted from a report by Georges et al.^61^ In short, 4-VBC (10 mL, 0.07 mol), St (27 mL, 0.24 mol), TEMPO (0.125 g, 0.8 mmol), and BPO (0.065 g, 0.27 mmol) were added to a 100 mL Schlenk flask. Three freeze–pump–thaw cycles were done, and then the mixture was stirred at 123 °C for 36 h under an argon atmosphere. The product was cooled to RT and dissolved in CH_2_Cl_2_. Finally, the polymer was precipitated into excess methanol. The white solid obtained was dried overnight in a vacuum at room temperature. The molar percentage of VBC in the copolymer [VBC] was determined by ^1^H NMR and found to be 24.8%.^62^ GPC analysis (THF): weight-average molecular weight (MW) = 46 kDa and polydispersity index (PDI) = 1.4 (Figure S2).

### Amination and Crosslinking of P(St-*co*-VBC) Membrane

P(St-*co*-VBC) (1.5 g) was dissolved in toluene (5 mL) and stirred until completely dissolved. Then, the solution was poured on a plate and the solvent evaporated at RT. The remaining film was soaked in excess of TEPDA for 72 h at 50 °C. The desired crosslinked P(St-*co*-VBC)-TEPDA (IEC = 1.30 mmol g^–1^ in Cl^–^ form) was obtained after the removal of the diamine by evaporation at room temperature.

### Hydroxide Conductivity Measurement Method

The *ex situ* method for measuring the true OH^–^ conductivity of AEMs was reported recently by Dekel et al.^58,63^ In summary, AEMs were first converted into their bicarbonate (HCO_3_^–^) form by soaking them in a NaHCO_3_ aqueous solution for two days and washing with DI water. Then, the AEMs were placed in a four-electrode cell (MTS 740, Scribner Associates, Inc.) to measure the anion conductivity. A 100 μA direct current (Ivium-n-Stat, Ivium Technologies) was applied through the external electrodes to the membrane under a continuous 500 cm^3^ min^–1^ nitrogen flow (99.999% N_2_) at 40 °C and 90% relative humidity (RH) to electro-generate the OH^–^ anions.

### Preparation of Water-Free Hydroxide Solution

Water-free potassium hydroxide in 18-crown-6 (CE/KOH) salt was prepared as previously reported.^35,46−48^ The solution was prepared by weighing the CE/KOH salt in a glovebox and dissolving it in anhydrous DMSO.

### Kinetics Studies and Measurement Method

For experiments under anhydrous conditions (λ = 0): CE/KOH (0.452 g, 1 mmol OH^–^) solution was dissolved in 1.2 mL of DMSO in a vial. DMSO was chosen as the solvent, given the high solubility of CE/KOH in it. The OH^–^ concentrations were found to be 0.5 M by titration. After mixing the solution on a shaker, a piece from the AEM under test (*ca*. 15 mg) was added to the vial to immerse in the CE/KOH solution at room temperature. After specific time points, a small sample from the AEM was cut off and taken out of the glovebox for analysis. For experiments with aqueous KOH, an aqueous solution of 0.5 M KOH was prepared, and the AEM under test (*ca*. 15 mg) was added to the vial and immersed in the solution at room temperature. Samples of the AEM were cut off and removed every few hours. Before characterization, all membrane samples were thoroughly washed with ultrapure water (UPW, 18 MΩ·cm) for 48 h. The samples were then converted to the chemically stable Cl^–^ form by immersion in aqueous KCl (1 M) for 72 h with three changes of KCl solution during this period. Finally, the membranes were soaked again in UPW for 48 h. All of the membranes, in their chloride form, were analyzed in a confocal Micro-Raman (LabRAM HR Evolution, 532 nm laser) or in an ATR-FTIR instrument (Bruker Tensor 27). The confocal Raman analysis was focused on the membrane surfaces, while in ATR-IR, the sample was analyzed. In addition, the membranes were analyzed for IEC using the Mohr titration method (described previously).^64^

## Results and Discussion

We previously demonstrated that BTMA and BTEA salts, as well as soluble AEMs, decompose rapidly in the presence of OH^–^ at room temperature as the water content decreases. This is because the nucleophilicity and basicity of the hydroxide ion increase as the number of water molecules solvating decreases.^65^ These results imply that the current aqueous alkali *ex situ* tests using highly hydrated alkali solutions used to measure AEM stability may provide false-positive stability results, causing anion-conducting polymers to appear more alkali-stable than they really are.^35,48^ In this study, our protocol combines an aggressive alkaline environment and low hydration level, for the first time to test nonsoluble and crosslinked AEMs. We aim to determine the significance of the interaction of these two effects on the chemical stability of QAs in polymers. For this study, two RG-AEMs are tested: ETFE-TEA and ETFE-TMA, using low and high hydration levels at room temperature.

The chemical structures of these AEMs are shown in Figure 1a. As reported in our previous studies, ETFE-TEA AEM is expected to decompose by Hofmann elimination (E2) degradation reactions because of the presence of the ethyl substituents on the covalently bound BTEA groups (Figure 1b). Such elimination reactions cannot occur with BTMA groups, which decompose *via* nucleophilic attack (S_N_2).^46^ First, both ETFE-TEA and ETFE-TMA AEMs were tested at room temperature using 0.5 M OH^–^ in water-free DMSO (λ = 0). The intermediate recording Raman spectra of both membranes are presented in Figure 1c,d.

The degradation of ETFE-TEA was calculated from the change in the area of the 1100 cm^–1^ Raman band (C–N vibration from the triethylammonium group, see the SI), normalized to the area of the 833 cm^–1^ band (C–F stretch from the ETFE backbone)^59^ during 14-day treatment in harsh alkaline conditions (Figure 1c). Calculations indicate 97, 89, 73, and 72% retention in the peak area after 1, 3, 7, and 14 days, respectively (Figure 1e).

The degradation of ETFE-TMA was estimated by changes in the area of the band at 753 cm^–1^ (C–H vibration of the trimethylammonium group)^55^ and the band at 833 cm^–1^, which relates to the ETFE backbone.^59^ In these water-free alkaline conditions, the peak area retention was 94, 89, and 85% after 7, 21, and 49 days, respectively.

As seen in Figure 1d, the band at 1180 cm^–1^ (corresponding to the formation of C–O bonds in benzyl alcohol groups) increases with time. The IEC retention after 7 days in these test conditions was 93 and 73% for ETFE-TMA and ETFE-TEA, respectively (Table 1), which is in good agreement with the Raman analysis data. Finally, the degraded half-lives were calculated for both RG-AEMs. Assuming a pseudo-first-order reaction (since the OH^–^ is in large excess), the corresponding half-lives of ETFE-TEA and ETFE-TMA were found to be 23 and 173 days, respectively (Figure 1e and Table 2). The experimental degradation data shown in Figure 1e was fitted to the linear equation: ln[QA] = ln[QA]_0_ + *k*·*t*, where *k* is the degradation rate constant, and [QA] and [QA]_0_ are the QA concentration at a given time *t* and initial concentration, respectively. While a decrease in rate is seen in the final point, the line shows a good linear fit, supporting the pseudo-first-order kinetics is adequate (Figure S4). The corresponding half-lives were calculated using the resulting relationship: .

**Table 1:** AEMs Degradation as Measured by Changes in IEC, Raman Data, ATR-FTIR Data, and True OH^–^ Conductivity after 7-Day Immersion in 0.5 M OH^–^ DMSO Solution (λ = 0)^a^

	remaining QA by IEC				
AEM	aqueous 0.5 M KOH (λ > 28)	0.5 M OH^–^ DMSO (λ = 0)	remaining QA by Raman^b^	remaining QA by ATR-FTIR^b^	relative conductivity^c^
ETFE-TEA	92%	73%	(73 ± 8) %	NA^e^	30%
ETFE-TMA	>99%	93%	(94 ± 4) %	NA^e^	87%^d^
PS-TEA	87%	55%	NA^e^	9%	NA^e^

a The IEC change after soaking the AEMs for 7 days in aqueous 0.5 M KOH (λ ≈ 26) is also reported. Errors are standard deviations calculated from *n* = 3 independent measurements.

b Calculated by changes in peak area ratio.

c True OH^–^ membrane conductivity of AEMs at a temperature of 40 °C and 95% RH.

d 87% remaining after 28 days to obtain measurable change.

e Measurement is not available.

**Table 2:** Degradation Rate Constants and Calculated Half-Lives of ETFE-TEA and ETFE-TMA in 0.5 M OH^–^ DMSO Solutions (λ = 0) at Room Temperature

	rate constant *k* [d^–1^]	half-life [d]
ETFE-TEA	0.030	23
ETFE-TMA	0.004	173

To conclude, ETFE-TMA is clearly more stable than ETFE-TEA under the same conditions, which follows the previously reported trend with solubilized BTMA- and BTEA-based salts and PPO-based ionomers.^35,48^ The *ex situ* results of the ETFE-TMA in this work are in the same order as its *in situ* reported lifetime in an AEMFC test,^66^ during which there was very little voltage degradation for more than 100 h in the absence of Pt-based catalysts.

**Figure 2:**
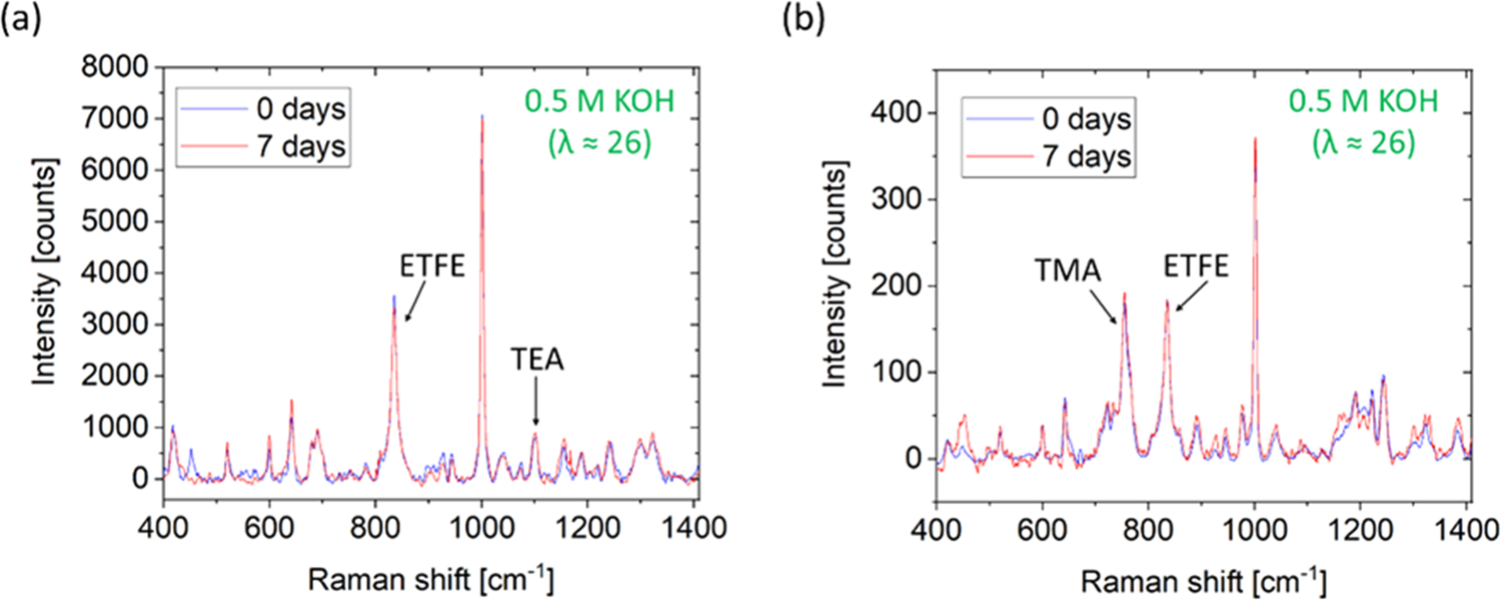
Raman spectra of (a) ETFE-TEA and (b) ETFE-TMA immersed in aqueous 0.5 M KOH solution (λ ≈ 26). All experiments were conducted at room temperature.

Given most of the reported stability tests in the literature were carried out in aqueous solutions, additional AEM stability testing was also conducted using aqueous 0.5 M KOH solutions (λ ≈ 26). The Raman data for both RG-AEMs are presented in Figure 2. No significant degradation was observed for ETFE-TEA (Figure 2a) and ETFE-TMA (Figure 2b) after 7 days of the test. According to Table 1, while comparing the changes in IECs when using both this aqueous alkali and water-free alkali (0.5 M CE/KOH), the difference between aqueous alkali degradation and λ = 0 alkali degradation is greater for ETFE-TEA than ETFE-TMA.

**Figure 3:**
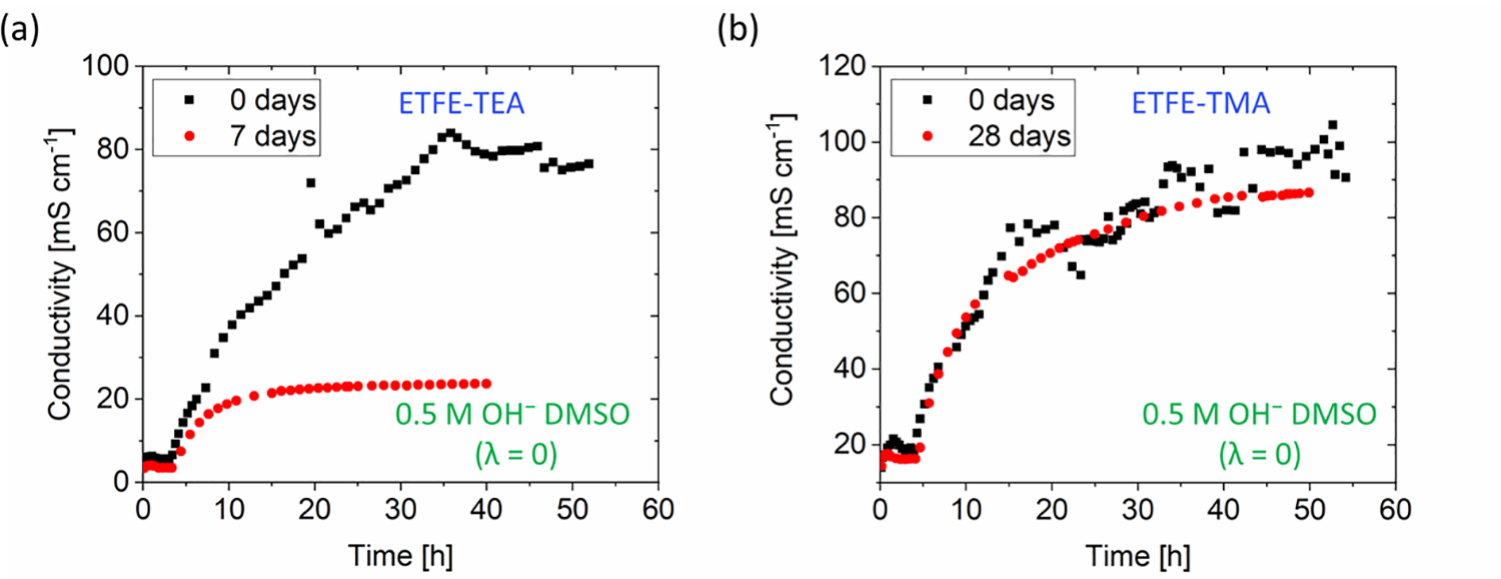
Changes in the conductivities of (a) ETFE-TEA and (b) ETFE-TMA before and after alkali stability testing at λ = 0 in 0.5 M OH^–^ DMSO solutions. The *x*-axes indicate the time that a 100 μA direct current was applied to convert the HCO_3_^–^ form RG-AEMs to their OH^–^ forms. Test conditions remained constant with flowing N_2_ (99.999%) at 90% RH and 40 °C.

To support the differences between our low hydration alkaline degradation measurements, the true OH^–^ ion conductivity^67^ for both RG-AEMs after 7 days soaking in CE/KOH solution was measured (Table 1 and Figure 3). As expected, the OH^–^ conductivities of ETFE-TEA rapidly stabilized at lower values after 7 day degradation, supporting the more extensive degradation of ETFE-TEA compared to ETFE-TMA (even when the latter is degraded under the same conditions for an extended 28 days). Note: the OH^–^ conductivity of 97 mS cm^–1^ for ETFE-TMA at 40 °C and RH = 90% exceeds the previously reported 60 mS cm^–1^ at a higher 95% RH at 40 °C,^68^ due to the more rigorous CO_2_ removal used in this later study.^69^

**Figure 4:**
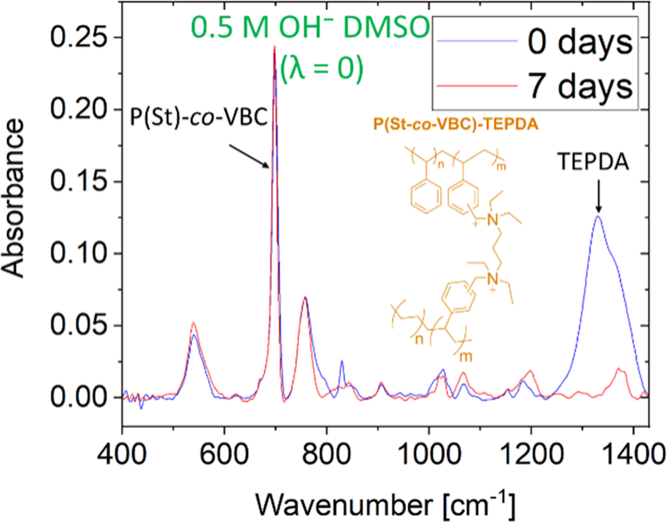
ATR-FTIR spectra of P(St-*co*-VBC)-TEPDA before and after degradation in λ = 0 in 0.5 M OH^–^ DMSO solutions at room temperature.

Finally, we also tested the new protocol on a crosslinked P(St-*co*-VBC)-TEPDA (structure in Figure 4). Due to the presence of β hydrogens, P(St-*co*-VBC)-TEPDA is expected to decompose by Hofmann elimination (E2), similarly to ETFE-TEA. Initially, we tried to monitor the decomposition of P(St-*co*-VBC)-TEPDA by Raman spectroscopy; however, no changes were seen over 14 days (Figure S5). Therefore, the degradation of P(St-*co*-VBC)-TEPDA was measured using ATR-FTIR, where more significant spectral changes were observed (Figure 4). The degradation of P(St-*co*-VBC)-TEPDA after 7 days was calculated using the area ratio between the bands at 1330 cm^–1^ (C–N vibration in the TEPDA groups) and 690 cm^–1^ (C–H vibration in the PS-*co*-VBC backbone, see Figure S6). The decrease in the ratio between the two band intensities is severe, indicating that only 9% of the original structure of P(St-*co*-VBC)-TEPDA remains after 7 days immersion in dry CE/KOH (Figure 4 and Table 1). Although P(St-*co*-VBC)-TEPDA was analyzed by ATR-FTIR instead of Raman analysis, it clearly presents a significantly more severe degradation compared to both ETFE-based AEMs. In addition to the change in the backbone, it is important to take into consideration that this functional group has two ammonium cations nearby, increasing the electron-withdrawing effects and further accelerating the E2 mechanism.^15,70^

## Conclusions

Herein, we describe the extension of a meaningful *ex situ* method for the measurement of the alkaline stability of insoluble anion-exchange membranes (AEMs) in a low hydration, high-pH environment. At low hydration levels, the alkali decomposition processes are accelerated (compared to measurements in hydrated environments), especially for QA groups that are able to decompose *via* Hofmann elimination. Under such measurement conditions, the undersolvated OH^–^ ions have increased basicity and nucleophilicity, leading to faster reactions with the QA groups. This technique allows for a practical, simple, and rapid *ex situ* study of the alkaline stabilities of AEM chemistries in an environment that mimics in *operando* environment inside fuel cells and water electrolyzers (in dry cathode operation mode), without the use of complex *in situ*/*operando* studies. These findings strongly emphasize the importance of measuring the stability of AEMs using a protocol that controls the solvation sphere of the OH^–^ anions. The methodology describes more tightly the environment of an operating electrochemical device, so resulting data are critical to the development of AEMs for durable AEMFCs and AEMWEs. We strongly recommend adopting this as a standard method for measuring the alkali stability of nonsoluble and crosslinked AEMs.

## Supporting Information Available

Calculation of composition of the random copolymer P(St-*co*-VBC) and Raman spectra (PDF)

## Abbreviations

AEM: anion-exchange membranes
AEMFC: anion-exchange membrane fuel cells Fuel cells
AEMWE: anion-exchange membrane water electrolyzer
QA: quaternary ammonium
BTMA: benzyltrimethylammonium
BTEA: benzyltriethylammonium
PPO: poly(2,6-dimethylphenylene oxide)
ETFE: poly(ethylene-*co*-tetrafluoroethylene)
RG: radiation-grafted
IEC: ion-exchange capacities
GPC: gel permeation chromatography
NMR: nuclear magnetic resonance
VBC: 4-vinylbenzyl chloride
St: styrene
TEMPO: 2,2,6,6-tetramethyl-1-piperidinyloxy
BPO: benzoyl peroxide
TEPDA: *N*,*N*,*N*′,*N*′*-*tetraethyl-1,3-propanediamine
MW: weight-average molecular weight
PDI: polydispersity index
CE/KOH: water-free potassium hydroxide in 18-crown-6
UPW: ultrapure water
RH: relative humidity
