# Supplementary material for: Alkaline Stability of Anion-Exchange Membranes
Source: ACS Appl Energy Mater. 2023 Jan 9;6(2):1085–92. doi: 10.1021/acsaem.2c03689 (PMC10016746; doi:10.1021/acsaem.2c03689)
Supplement: Supplementary file 1 — ae2c03689_si_001.pdf [file ae2c03689_si_001.pdf]

Supporting Information for

Alkaline Stability of Anion Exchange Membranes

*Sapir Willdorf-Cohen[a], Avital Zhegur-Khais[a], Julia Ponce-González[b], Saja Bsoul-Haj[a], John R. Varcoe[b, *], Charles E. Diesendruck[c, d, *], Dario R. Dekel[a, d, *]*

[a]*The Wolfson Department of Chemical Engineering, Technion – Israel Institute of Technology, Haifa 3200003, Israel.*

[b]*School of Chemistry and Chemical Engineering, University of Surrey, Guildford GU2 7XH, United Kingdom.*

[c]*Schulich Faculty of Chemistry, Technion-Israel Institute of Technology, Haifa 3200003, Israel.*

[d]*The Nancy & Stephen Grand Technion Energy Program (GTEP), Technion – Israel Institute of Technology, Haifa 3200003, Israel.*

* Corresponding authors

E-mail addresses: j.varcoe@surry.ac.uk (John R. Varcoe), charles@technion.ac.il (C.E. Diesendruck), dario@technion.ac.il (D.R. Dekel)

1. Calculation of composition of the random copolymer P(St-co-VBC)

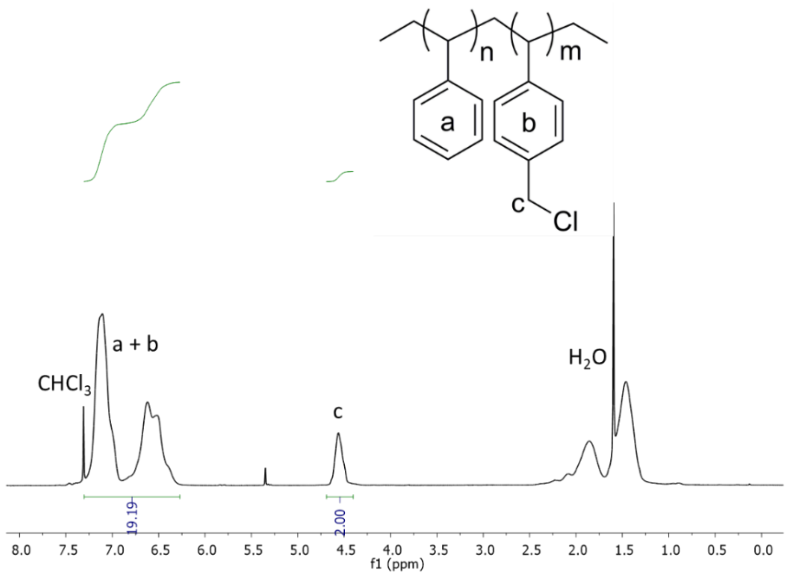

Figure S1. *1H NMR spectrum of the random copolymer P(St-co-VBC).*

Molar ratio of the monomeric units F = [St]/[VBC] in random copolymers P(St-*co*-VBC) was calculated from ^{1}H NMR spectra according to the following equations[1]:

$$F = \frac{[St]}{[VBC]} = \frac{(I_{(a+b)} - 2 \times I_c)/5}{I_c/2}$$

where $I_{(a+b)}$ is the integral value of the signals at 6.3–7.45 ppm attributed to the aromatic protons, and I_c is the integral value of the signal at 4.6 ppm attributed to the protons of chloromethyl group (–CH_2Cl) in VBC unit.

Since: $I_{(a+b)} = 19.19\,ppm, I_c = 2\,ppm$

The value of F is:

$$F = \frac{[St]}{[VBC]} = 3.038 \rightarrow [St] = 3.038 \times [VBC]$$

While [St] and [VBC] are molar parts of St and VBC units in a copolymer, respectively and therefore: $[St]+[vbc]=1$.

Finally, the molar composition of the random copolymers was calculated by solving the following equation:

$$\%[VBC] = \frac{[VBC]}{[VBC]+[St]} = \frac{[VBC]}{[VBC]+3.038\times[VBC]} \times 100\% = 24.8\%$$

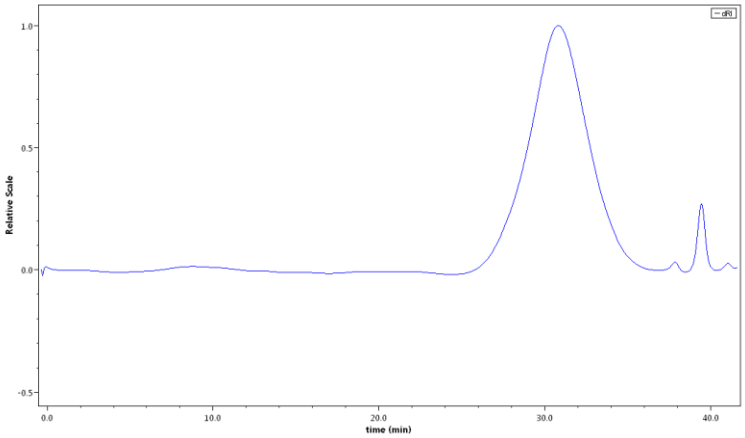

Figure S2. *THF-GPC (signal from differential refractive-index detector) of linear precursor P(St-co-VBC), Mw of 46 kDa.*

2. Raman spectra

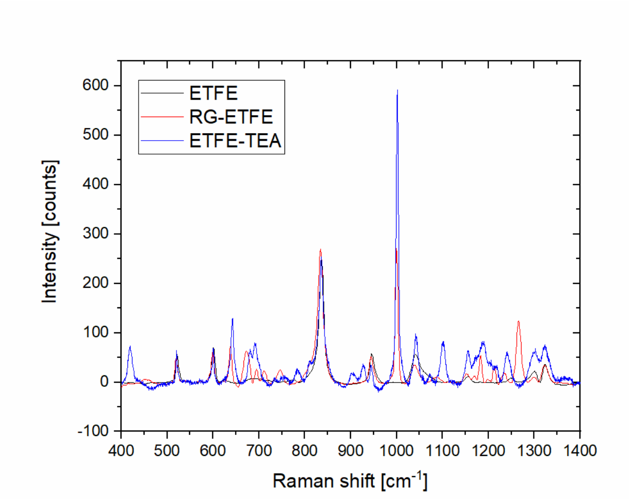

Figure S3. *Raman spectra of ETFE, RG-ETFE and ETFE-TEA. Data of ETFE and RG-ETFE were reported Varcoe and co-authors,*[2] *and the peaks at 1100 and 690 cm^{-1} are diagnostic of triethylammonium groups.*

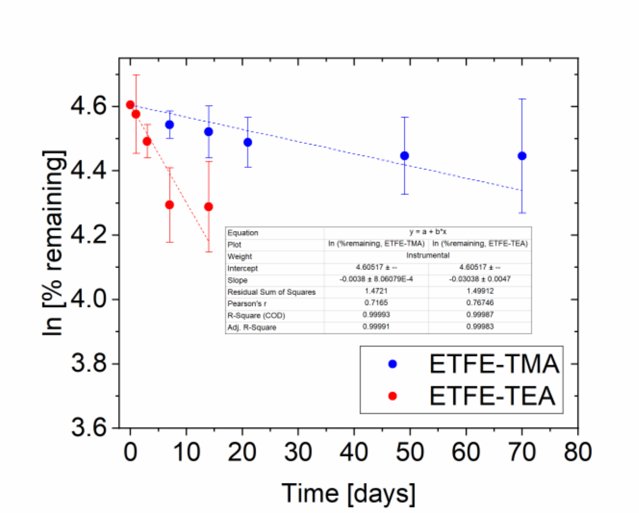

Figure S4. *Logarithmic QA decay in ETFE-TMA and ETFE-TEA as a function of time measured by Raman, when tested in 0.5 M OH^- DMSO solutions (λ=0). Error bars represent standard deviations from three measurements.*

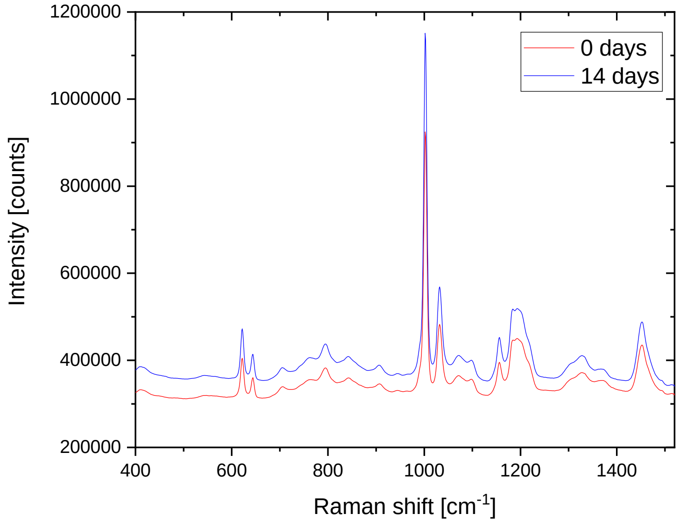

Figure S5. *Raman spectra of P(St-co-VBC)-TEPDA before and after degradation in λ = 0 in 0.5 M OH^- DMSO-d_6 solutions at room temperature. Provide as an evidence that there is no change in Raman peaks.*

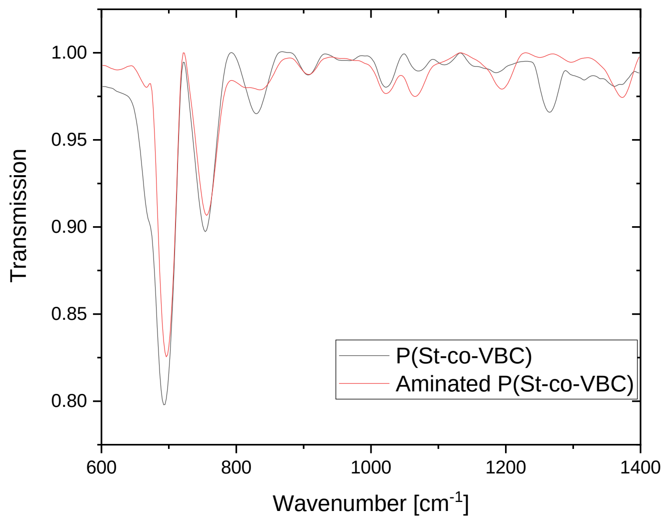

Figure S6. *Raman spectra of pristine and aminated P(St-co-VBC).*

3. References

(1) Jonikaite-Svegzdiene, J.; Kudresova, A.; Paukstis, S.; Skapas, M.; Makuska, R. Synthesis and Self-Assembly of Polystyrene-Based Diblock and Triblock Coil-Brush Copolymers. *Polym. Chem.* **2017**, *8* (36), 5621–5632. https://doi.org/10.1039/c7py01335c.

(2) Ponce-González, J.; Whelligan, D. K.; Wang, L.; Bance-Soualhi, R.; Wang, Y.; Peng, Y.; Peng, H.; Apperley, D. C.; Sarode, H. N.; Pandey, T. P.; Divekar, A. G.; Seifert, S.; Herring, A. M.; Zhuang, L.; Varcoe, J. R. High Performance Aliphatic-Heterocyclic Benzyl-Quaternary Ammonium Radiation-Grafted Anion-Exchange Membranes. *Energy Environ. Sci.* **2016**, *9* (12), 3724–3735. https://doi.org/10.1039/C6EE01958G.
